# Inoculation against Cholera

**Published:** 1892-10

**Authors:** 


INOCULATION AGAINST CHOLERA.—Haffkine having found by recent experiments that anti-cholera vaccine prepared after Pfeiffer's methods had the same action on various sorts of animals, ventured to try the inoculation himself. He injected subcutaneously in his left side a larger dose than that used in animals, of the so-called first kind of anti-cholera vaccine. The indisposition which followed lasted twenty-four hours, and consisted of a rise of temperature of 1 degree C., with headache, dryness of the mouth, and clouding of the urine, with no disturbance of the digestive tract. Locally there was pain, slight swelling, and glandular enlargement. The pain lasted five days. The swelling gradually diminished and disappeared on the ninth day. Six days after the first inoculation, Haffkine had the other side inoculated with the strengthened cholera virus (vaccine No. 2). Again there was a rise of temperature and local pain, but no swelling. The general condition was normal in twenty-eight hours, and the pain disappeared in three days. There were no digestive disturbances. The inoculation was tried in three other persons with similar results. In one of these cases a slight diarrhœa, which had lasted for several days, stopped the day after the first injection. The inoculation of both kinds of anti-cholera vaccine, the protective power of which in animals has been experimentally proved, is harmless to men, and Haffkine entertains the hope that six days after inoculation with this vaccine, the human organism will have obtained complete immunity against every cholera infection.—*Ex.*

